# Molecular Engineering L‐Aspartate‐Alpha‐Decarboxylase to Enhance Catalytic Stability and Performance

**DOI:** 10.1002/open.202400236

**Published:** 2024-10-25

**Authors:** Zihan Liu, Yiheng Liu, Qixuan Jiang, Haijun Xu, Luo Liu

**Affiliations:** ^1^ Beijing Bioprocess Key Laboratory Beijing University of Chemical Technology Beijing 100029 PR China

**Keywords:** Computational analysis, Directed evolution, Enzyme engineering, L-aspartate-alpha-decarboxylase, Mutation combination, β-alanine

## Abstract

L‐aspartate‐alpha‐decarboxylase (ADC) catalyzes the decarboxylation of L‐aspartate to produce β‐alanine, which is the decisive step in the biosynthesis of β‐alanine. However, the low catalytic stability and efficiency of ADC limit its industrial applications. In this study, a variant of ADC from *Bacillus subtilis* were used as a starting point for engineering. After constructing a random mutagenesis library by error‐prone PCR, followed by high‐throughput screening,four substitutions (S7 N, K63 N, A99T, and K113R) were identified. By screening saturation mutagenesis libraries on these positions and computational analysis, two recombined variants N3(S7 N/K63 N/I88 M/A99E/K113R/I126*) and Y1(S7Y/K63 N/I88 M/A99E/K113R/I126*) with improved performance were obtained. Compared to the wild type, the catalytic efficiency and catalytic stability of the best two variants were enhanced up to 95 %(variant N3) and up to 89 %(variant Y1), respectively. In addition, Y1 exhibited 3.37 times improved half‐life and 2‐fold improved total turnover number. Hydrophilicity analysis and molecular dynamics (MD) simulation revealed that the increased hydrophilicity and steric hindrance of key amino acid residues would affect the catalytic activity and stability. The improved catalytic performance of the variants could be attributed to their enhanced binding capacity to the substrate within the active pocket and the alleviation of mechanism‐based inactivation.

## Graphical abstract

Four substitutions of L‐aspartate‐alpha‐decarboxylase were screened out by directed evolution. By saturation mutagenesis and computational analysis, two recombined variants with improved catalytic performance were obtained. The improved catalytic performance of the variants could be attributed to enhanced binding capacity to the substrate within the active pocket and the alleviation of mechanism‐based inactivation.

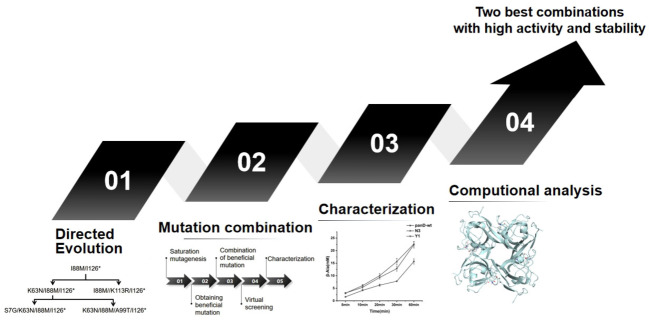

## 1 Introduction

As the only β‐type amino acid in nature, β‐alanine plays a crucial role in the synthesis of pantothenic acid, coenzyme A, acyl carrier proteins in organisms.[^1^, ^2^, ^3^] β‐alanine is extensively utilized in medicine, chemical industry, food, environment and other fields, especially in the synthesis of calcium pantothenate^[4]^ and carnosine,^[1]^ serving as a vital raw material for industrial synthesis. L‐aspartate‐alpha‐decarboxylase (ADC) catalyzes the decarboxylation of L‐aspartate to synthesize β‐alanine.^[5]^ Currently, ADC are predominantly categorized into two types based on their cofactors, one using PLP(pyridoxal phosphate) and the other using pyruvate. Despite the widespread presence of ADC in prokaryotes and insects,^[6]^ it is only the pyruvoyl‐dependent ADC sourced from *Escherichia coli*, *Bacillus subtilis*, and *Corynebacterium glutamicum* that currently hold the potential for industrial‐scale production.

Pyruvoyl‐dependent ADC, during translation initiation, initially generates an inactive pro‐protein(π protein). Subsequently, a protein hydrolysis occurs at the Gly24‐Ser25 site, resulting in the cleavage of the ester bond to produce the C‐terminal hydroxyl‐bearing β‐subunit (2.8 kDa) and the N‐terminal acyl group‐bearing α‐subunit (11 kDa).[^7^, ^8^] Further conversion of the serine at the N‐terminus of the α‐subunit into an pyruvyl group grants the protein its activity. The catalysis of L‐aspartate decarboxylation by pyruvoyl‐dependent ADC involves four steps: the pyruvyl group generated from Ser25 forms a Schiff base with the L‐aspartate; the Schiff base undergoes decarboxylation to release carbon dioxide, forming an enol intermediate; the enol intermediate is attacked by a proton generated from the phenol hydroxyl group of Y58, forming an imine structure; the imine structure cleaves to yield β‐alanine, thereby regenerating the pyruvyl group.[^9^, ^10^] However, if the proton attacks the imine structure instead of the enol structure, it results in a change in the position of the imine structure, ultimately causing the conversion of the pyruvyl group into a alanyl group and rendering it inactive.[^9^, ^10^]

Low catalytic stability and activity due to mechanistic inactivation are major problems with ADC. To address these problems, currently, the augmentation of ADC activity and catalytic stability is primarily accomplished through the construction of variant libraries using site saturation mutagenesis,^[11]^ followed by subsequent screening processes. Pei^[12]^ addressed the deactivation phenomenon observed in the catalytic process of BsADC and introduced the concept of catalytic stability. They proposed that strengthening the correct protonation process via molecular biology methods might inhibit incorrect protonation. Subsequently, they screened variants I88 M and V68I/L127*, showing notable enhancements in enzyme activity (18 %–22 %) and stability (29 %–64 %). Zhang^[13]^ conducted a comparative analysis of ADC derived from *Escherichia coli*, *Corynebacterium glutamicum*, and *Bacillus subtilis*, finding that ADC from *Bacillus subtilis* exhibited superior catalytic activity and stability. They identified the variant E56S, which showed a 55 % increase in enzyme activity and a 40 % improvement in catalytic stability compared to the wild type. Qian^[14]^ determined the enzymatic properties of the variant Q5 (I46 V/I88 M/K104 S/I126*) and found that increasing the distance between the proton donor Y58 and the active site S25 could increase the difficulty of incorrect protonation, reduce the flexibility of certain regions, and thus enhance the catalytic stability of BsADC. However, current studies have offered a less comprehensive analysis of the inactivation mechanism, as well as the increased enzyme activity and catalytic stability of the dominant variants. Furthermore, there are no rational strategies currently available for modifying the activity or stability of ADC.

This study aimed to improve the activity and catalytic stability of BsADC through enzyme engineering campaign. Random mutagenesis and high‐throughput screening were carried out to identify amino acid residues with significant impacts on enzyme activity and catalytic stability. Substitution of amino acid residues often affects the hydrophilicity, hydrogen bonding, steric hindrance and structural flexibility of the enzyme. By screening saturation mutagenesis libraries on these positions and computational analysis, the best recombined variants were obtained and exhibited higher catalytic activity and stability. Structural analysis revealed that increasing the hydrophilicity of key residues in the active pocket and the distance between the proton donor and the active site could improve the catalytic performance.

## Experimental

### Strain, Medium, Culture Conditions

The target gene *panD* (Gene ID: 939033) was inserted to the plasmid pET‐30a(+) with *Nde*I and *Hind*III as the restriction enzyme sites, resulting in the recombinant plasmid pET‐30a(+)‐*panD*. The recombinant plasmid was transformed into *Escherichia coli* BL21(DE3) cells(Weidi, Shanghai, China) and cultured in LB medium (10 g/L tryptone, 10 g/L sodium chloride, 5 g/L yeast extract, pH 7.2–7.4) containing 50 μg/L kanamycin at 37 °C with shaking at 220 rpm.

### Construction of Randomized Variant Libraries and High‐throughput Screening Methods

To screen for beneficial variants of BsADC, the variant template I88 M/I126* was selected, and random mutagenesis library was constructed by using error‐prone PCR (Table S2) with the universal primers T7 and T7term (Table S1). QuickMutation™ Razndom Mutagenesis Kit from Beyotime (Shanghai, China) was utilized. When constructing random variant library, the mutation rate was controlled to be approximately 1 % by adjusting the amount of template added, the annealing temperature and the amount of other components in the PCR system. The agarose gel electrophoresis results showed that the error‐prone PCR bands were of the correct size, and the sequencing results showed that the mutation rate was in accordance with the requirements. Saturation mutagenesis was carried out using NNK primers (Table S2). Following digestion with DpnI enzyme to remove the template plasmid, the PCR product was purified by plasmid purification kit(Omega, America). Subsequently, it was ligated to the plasmid pET‐30a(+) through homologous recombination and circularized. The recombinant plasmid was then transformed into *Escherichia coli* BL21(DE3), and the recombinant cells were inoculated on LB agar plates containing agar and 50 μg/L kanamycin.

Single colonies were picked and inoculated into a 96‐well plate containing kanamycin‐LB medium and cultured at 37 °C with shaking at 750 rpm. When the optical density (OD_600_) reached 0.6–0.8, isopropyl‐β‐D‐1‐thiogalactopyranoside (IPTG) was added to a final concentration of 0.2 mM, and the culture was induced for 12 h at 30 °C. The cell pellet was obtained by centrifugation at 5000×g for 15 min, followed by resuspension in DMSO‐PBS buffer (5 % DMSO, pH 7.0). 50 μL of cell suspension was taken and mixed with 100 μL of Asp‐PBS buffer (50 mM, pH 7.0), and the reaction was carried out at 37 °C for 1 h. In a 96‐well plate, 15 μL of reaction mixture, 135 μL of sodium borate buffer (0.2 mM, pH 9.5), 2.5 μL of 1,4‐diacetoxybenzene‐methanol solution (10 mg/mL), and 2.5 μL of mercaptoethanol‐ ethanol solution (5.7 mg/mL) were added. All chemicals utilized were of analytical grade and purchased from Macklin (Shanghai, China). Fluorescence measurements were taken every 4 min using an excitation wavelength of 355 nm and an emission wavelength of 445 nm.[^12^, ^15^] The highest fluorescence peak within 2 h was determined for screening beneficial variants. Fluorescence value was measured by EnSpire Multimode Plate Reader(PerkinElmer, America).

### Protein Expression and Purification

By adding a His6 tag at the C‐terminus of BsADC, the recombinant protein was expressed as a His‐tag fusion protein in *Escherichia coli* BL21(DE3). The recombinant cells strain was cultured in kanamycin‐LB medium(100 mL) at 37 °C and 220 rpm. Upon reaching an OD 600 of 0.6–0.8, 0.2 mM IPTG was added, and the culture was induced for 12 h at 30 °C. The cell pellet was obtained by centrifugation at 8000×g for 10 min. After resuspension in PBS buffer, ultrasonic disruption was performed using an ultrasonic disruptor. Cell debris was eliminated through centrifugation (12,000 × g, 40 min) at 4 °C, and the resulting supernatant underwent filtration using a 0.45 μm filter membrane. Subsequently, purification was achieved using a Ni‐His binding column, leading to the isolation of pure enzyme. The imidazole concentrations in the wash buffer and elution buffer were 30 mM and 300 mM, respectively. Protein concentration was determined using the Coomassie Brilliant Blue method. The SDS‐PAGE picture of the purified protein shows that the purified protein can be used for the reaction (Figure S1).

### Determination of Enzyme Activity and Catalytic Stability

In the reaction system, 1 mL of PBS buffer containing Asp (40 mM, pH 7.0) and 25 μg of purified enzyme were added. The reaction was carried out at 37 °C and 200 rpm for 5 min. Then 500 μL of the reaction mixture was mixed with 250 μL of triethylamine‐acetonitrile (1 mol/L) and 250 μL of PITC‐acetonitrile to terminate the reaction and derivatize in the dark for 1 h. The production of β‐alanine was determined by HPLC(SHIMADZU, Japan). ADC activity units were defined as the amount of enzyme catalyzing the production of 1 μmol β‐alanine from L‐aspartate per minute at 37 °C.

The β‐alanine samples were derivatized with PITC and the concentration of β‐alanine was detected using HPLC. A C18 column was used. Mobile phase A was 80 % acetonitrile‐water and mobile phase B was 0.1 mol/L sodium acetate‐acetonitrile (97 : 3, pH 6.5). The detection conditions were set as follows: column temperature 40 °C, detection wavelength 254 nm. The assay program included a decrease in mobile phase B from 95 % to 65 % over 0–20 min, an increase from 65 % to 95 % over 20–30 min, and a constant concentration of mobile phase B from 30–35 min.

In order to elucidate the mechanistic inactivation and catalytic stability of the recombinant enzyme during the catalytic process, reactions were carried out at 37 °C and 200 rpm for 10 min, 20 min, 30 min, and 60 min. Following each reaction, the samples were derivatized for HPLC detection. A polynomial was fitted to the β‐alanine yield versus the reaction time, and the derivatives of the curves were used to calculate the instantaneous activity at different time points. The initial enzyme activity was defined as 100 %, allowing for the calculation of the residual activity at each time point.

### Determination of Kinetic Characterization

To determine the kinetic characterization Km and Vmax, different concentrations of L‐aspartic acid were added to the reaction system, and the reaction was carried out at 37 °C for 5 min. Then, 500 μL of the reaction solution was taken for derivatization for HPLC detection. The kinetic characterization Km and Vmax of the BsADC‐catalyzed process were calculated by Michaelis‐Menten equation, and kcat, catalytic efficiency (kcat/Km), and total number of conversions were calculated.

### MD Simulation and Structural Analysis

To better understand the reasons for the increased ability of the variant to catalyze the production of β‐alanine, molecular dynamics simulations were performed using YASARA(Supplementary Note), and structural analyses were conducted in conjunction with pymol. The amino acid sequence of L‐aspartate‐α‐decarboxylase from *Bacillus subtilis* was downloaded from NCBI (https://ncbi.nlm.nih.gov), and subsequently modeled by AlphaFold2 (https://alphafold2.com) and Swissmodel (https://swissmodel.expasy.org). The models were further scored by SAVESv6.0 (https://saves.mbi.ucla.edu) and the protein structures with the highest scores were selected for further analysis. Amino acid mutagenesis and calculation of ΔΔG were performed using YASARA combined with FoldX, followed by energy minimization. Molecular dynamics simulations of the wild type, variant N3, and Y1 were carried out for 30 ns at pH 7.0 and 303.15 K. The simulation snapshots were captured every 100 ps. The simulation script was ‘md run.mcr’, and the analysis script was ‘md analyse.mcr’. Additionally, the hydrophilicity analysis was calculated through the online platform Expasy (https://www.expasy.org).

## 2 Results and Discussion

### 2.1 Directed Evolution

**Figure 1:**
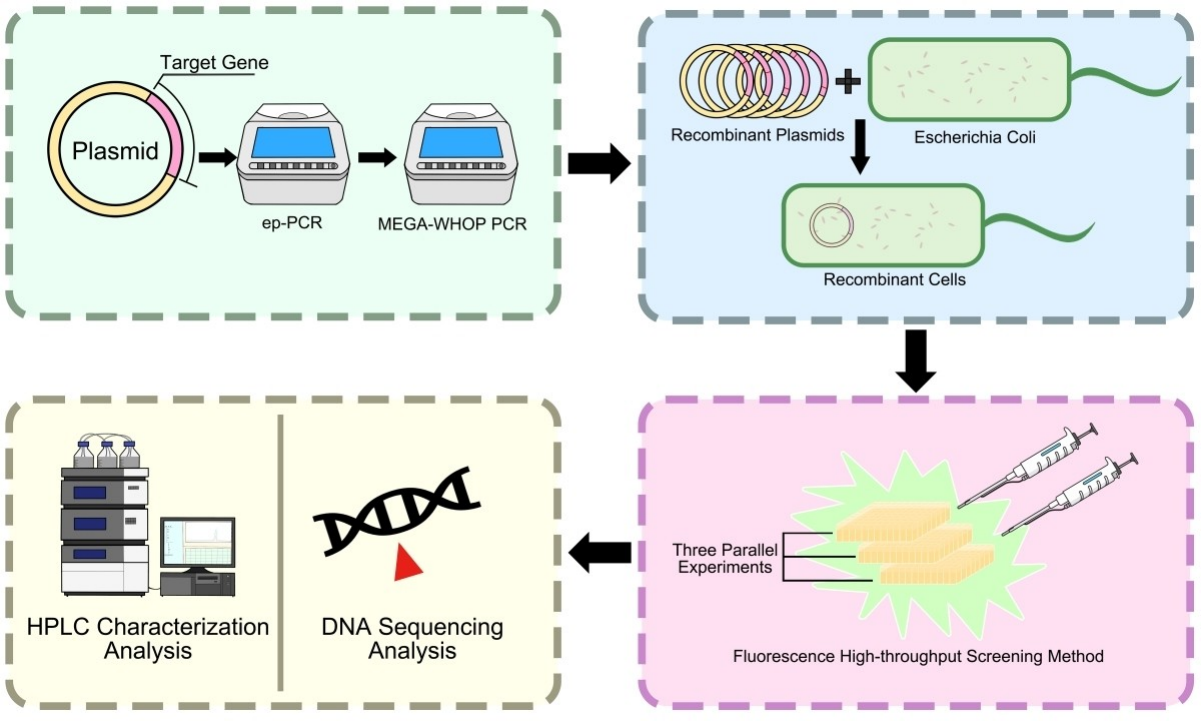
The equation of high‐throughput screening.

As substitution I88 M was reported in the literature with improved catalytic performance and C‐terminal deletion strategy can increase catalytic stability,[^12^, ^14^] therefore variant (I88 M/I126*) was selected as the template. Firstly, an ep‐PCR library was generated (>2000 clones) and the library was screened using fluorescence high‐throughput screening strategy (Figure 1). Then the genes encoding for the improved variants were sequenced, and the beneficial variants were further validated by HPLC. Two variants panD‐63 (K63 N/I88 M/I126*) and panD‐113 (I88 M/K113R/I126*) were identified with improved catalytic performance (Figure S2). Second round of directed evolution was based on variant panD‐63 which has better catalytic performance (Figure S3). After screening and sequencing, best two variants panD‐63‐7 (S7 N/K63 N/I88 M/I126*) and panD‐63‐99 (K63 N/I88 M/A99T/I126*) with improved catalytic performance were identified (Figure S2).

### 2.2 Mutation Combinations, Virtual Screening and Experimental Validation

**Figure 2:**
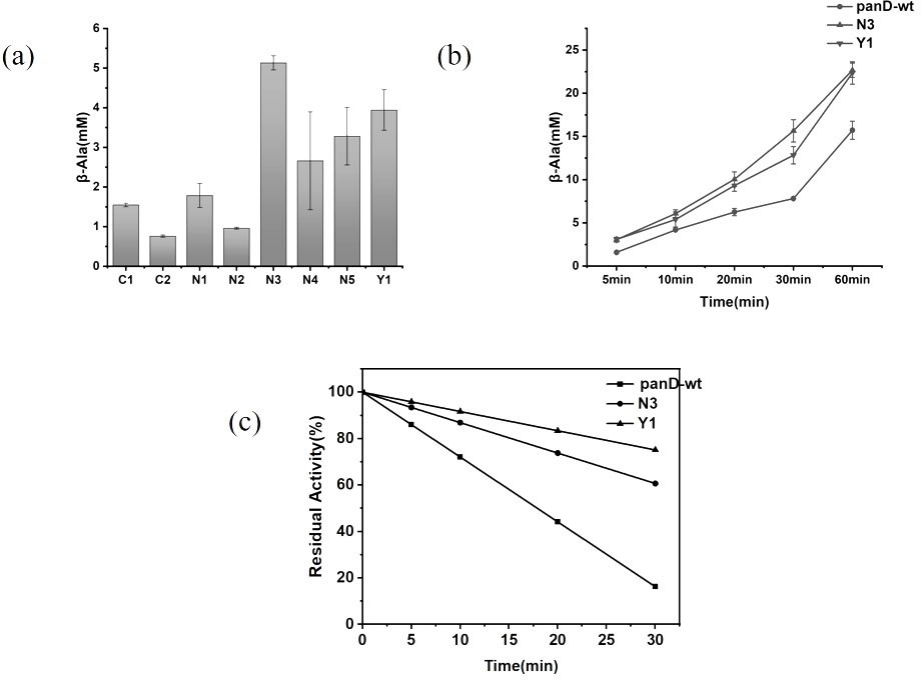
(a) β ‐alanine accumulation of different combinations. (b) β ‐alanine accumulation in different time periods. (c) residual activity in 30 min. The residual activity of BsADC is obtained by polynomial fitting and derivation calculations.

The variant panD‐63 was selected as the foundation for saturation mutagenesis at S7, A99, and K113. Subsequent high‐throughput screening and sequencing were conducted. It is noteworthy that the amino acid residues of the dominant variants obtained from the saturation mutation screen at these three sites showed a certain pattern. Among the variants at S7, A99 and K113 site, the more catalytically effective variants mutated to amino acid residues with larger side chains or more hydrophilic. Analysis of the BsADC structure revealed that S7 and A99 were situated in proximity to the active pocket, and K113 was positioned on the outer surface of the BsADC tetramer structure. Combining the experimental results and analysis, it was hypothesized that, at sites S7 and A99, increased steric hindrance and hydrophilicity may influence the conformation of the substrate pocket, while at site K113, the augmented hydrophilicity of the protein's surface may impact the physicochemical properties of the protein. Based on the above speculations, three site with different amino acid residues (S7 N, S7 C, S7Y, A99 V, A99E, A99T, K113R, K113E) were selected for combination. The ΔΔG‐fold values of different combinations were calculated by FoldX, and eight possible dominant mutation combinations were identified according to the CompassR‐guided recombination(Table 1).[^16^, ^17^, ^18^] Two mutation combinations N3 (S7 N/K63 N/I88 M/A99E/K113R/I126*) and Y1 (S7Y/K63 N/I88 M/A99E/K113R/I126*) with enhanced catalytic activity and catalytic stability were identified by experimental validation (Figure 2(a)).

**Table 1:** ΔΔG‐fold of the variants.

Serial number	Variants	ΔΔG(kcal/mol)
C1	S7C/K63N/A99E/K113R	−0.00256648
C2	S7C/K63N/A99T/K113R	−0.101781
N1	S7N/K63N/A99C/K113R	−0.202330
N2	S7N/K63N/A99E/K113E	−0.0948427
N3	S7N/K63N/A99E/K113R	−0.543583
N4	S7N/K63N/A99T/K113R	−0.388018
N5	S7N/K63N/A99V/K113R	−0.241144
Y1	S7Y/K63N/A99E/K113R	−0.121693

### 2.3 Enzyme Activity and Catalytic Stability of N3, Y1

Wild‐type(panD‐wt), N3 and Y1 variants of BsADC were expressed and purified, and the concentration of β‐alanine in the reaction system was determined by HPLC at different time points to calculate the catalytic efficiency and catalytic stability (Figure 2(b)). The data analysis revealed that both BsADC activity and catalytic stability were improved in N3 and Y1 compared to panD‐wt. Compared with panD‐wt, the catalytic efficiencies of N3 and Y1 were found to be significantly enhanced by 92.84 % and 94.58 % respectively at 5 min, which were much higher than other variants. Furthermore, when the reaction time reached 1 h, the substrate accumulation of N3 and Y1 increased by 44.24 % and 42.23 % respectively.

Smith,^[10]^ in studying the phenomenon of pyruvoyl‐dependent ADC inactivation, hypothesized that abnormal protonation of the intermediate imine structure during decarboxylation resulted in the conversion of the pyruvyl group to an alanyl group in the active center of the pyruvoyl‐dependent ADC, and thus the loss of catalytic inactivation. Further studies have suggested that this inactivation is a time‐dependent first‐order reaction. Pei^[12]^ first proposed the term “catalytic stability” to describe the mechanistic inactivation of pyruvoyl‐dependent ADC during catalysis. By comparing the product accumulation of panD‐wt, N3, and Y1 within 1 h, it was found that the amount of β‐alanine produced by catalytic decarboxylation of N3 and Y1 was higher than panD‐wt (Figure 2(b)). The instantaneous activity was obtained by polynomial fitting and derivation calculations, showing a better fit (Figure S4). Defining the initial enzyme activity without reaction as 100 %, the percentage of remaining activity for each time point for the initial enzyme activity was calculated, fitted and plotted as a catalytic stability curve. The active center of the enzyme was saturated in the presence of a far excess of substrate. The residual activity at 20 min was increased by 67.3 % and 89.11 % for N3 and Y1 respectively compared with the wild type (Figure 2(c)). The half‐lives of panD‐wt, N3, and Y1 were 17.91 min, 38.18 min, and 60.35 min, respectively. N3 and Y1 showed greatly improved catalytic stability and longer catalytic times compared with panD‐wt. It can be seen that variants N3 and Y1 slowed down the inevitable mechanistic inactivation during the catalytic process, but did not completely eliminate it. In the cell transformation experiment, the conversion rate of N3 and Y1 was over 95 % in 0.5 mol/L system and over 90 % in 1 mol/L system, which shows that these two mutation combinations have good application potential.

### 2.4 Kinetic Characterization of N3, Y1

The kinetic characterization of panD‐wt, N3, and Y1 variants of BsADC at 37 °C were investigated by determining the amount of β‐alanine accumulated by ADC at different substrate concentrations over 5 min. The Km and kcat values were calculated by plotting double inverse curves and the parameters of enzymatic properties were shown in the Table 2. Compared with panD‐wt, the kcat and kcat/Km of N3 and Y1 increased, indicating that the catalytic efficiency of the two variants was improved. Additionally, the Km of variant N3 decreased, indicating that the affinity to the substrate L‐aspartic acid was increased. It was speculated that the binding ability of the active center to the substrate was improved, thus improving the catalytic efficiency of BsADC. Furthermore, compared with panD‐wt, the total turnover number (TTN) of N3 and Y1 increased to 2.01‐fold and 2.03‐fold, respectively, which improved the catalytic life of the enzyme.^[19]^

**Table 2:** Characterization of panD‐wt, N3, Y1.

Variants	Km (mM)	kcat (s^−1^)	kcat/Km (s^−1^ mM^−1^)	Inactivation rate (s^−1^)	TTN
panD‐wt	3.69±0.33	4.25±0.45	1.15	0.0011	830.56
N3	3.22±0.15	8.29±0.17	2.57	0.0003	1676.30
Y1	3.82±0.29	7.26±0.22	1.90	0.0002	1685.46

*Date derived from Michaelis−Menten equation. All data shown were average values from measurements performed in triplicate.

### 2.5 Structural Analysis of Combinations

**Figure 3:**
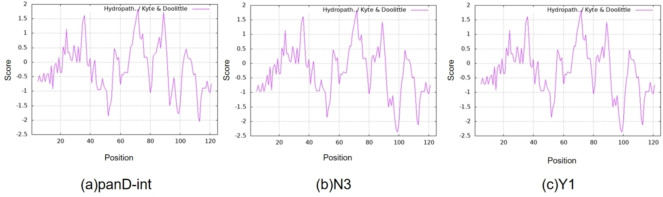
On‐line server calculates the hydrophilicity of amino acid residues. (a) panD‐int (b) N3 (c) Y1.

In order to exclude the influence of template mutation, panD‐int was selected for comparison. Hydrogen bonds play a crucial role in the combination of aspartate in the active pocket of BsADC and modifications of hydrophilicity may potentially affect this binding process. The hydrophilicity changes of variants were calculated through the Expasy online platform, with hydrophilicity being evaluated and scored according to the Kyte‐Doolittle scheme.^[20]^ A lower score indicates higher hydrophilicity, and when the score is less than −0.5, the amino acid is considered hydrophilic. Compared to panD‐int(Figure 3), substitution S7 N/S7Y, A99E, K113R led to increased hydrophilicity in their adjacent regions (M1‐H11, D95‐P103, N109‐M117). In region M1‐H11, it is important to note that the hydrophilicity scores of Lys9 in N3 and Y1 decreased from −0.378 to −0.678 and −0.433, respectively, indicating a significant increase in hydrophilicity. Previous studies suggested that Lys9 plays a key role in forming hydrogen bonds and binding with the substrate L‐aspartic acid during decarboxylation of L‐aspartic acid (Figure S5).^[21]^ It was speculated that the enhanced hydrophilicity of Lys9 improved its binding efficiency with L‐aspartic acid, facilitating the completion of the decarboxylation process. With respect to region D95‐P103 and N109‐M117, structural analysis revealed its location on the outer surface of the BsADC tetrameric structure. Proteins typically have hydrophobic side chains internally to maintain conformation, while hydrophilic side chains interact with water on the protein surface. This arrangement ensures the stability and conformation of the protein in an aqueous environment.[^22^, ^23^] Therefore, the increased hydrophilicity in the D95‐P103 and N109‐M117 region was inferred to improve the surface hydrophilicity of the BsADC tetramer, contributing to its stable conformation in the aqueous environment and thus enhancing the enzyme's stability in the catalytic system. In general, the increased hydrophilicity of M1‐H11 in the active pocket, particularly residue Lys9, enhanced the binding capacity of BsADC active center‐associated amino acid residues with the substrate aspartic acid, and improved the catalytic efficiency; the increased hydrophilicity of D95‐P103 and N109‐M117 in protein surface improved the catalytic stability.

To investigate the impact of the mutation sites on other amino acid residues and the overall conformation of BsADC, energy minimization and 30 ns molecular dynamics simulations were conducted for combinations by YASARA. Root mean square deviation(RMSD) is commonly used to study the overall conformational stability of protein backbones in dynamic environments.^[24]^ It was found that the average RMSD of K63 N decreased to 1.03703 within 30 ns (Figure S6(a)), indicating that this substitution increased the stability of the BsADC structure. Analysis of hydrogen bonds within the tetramer shows that the number of hydrogen bonds of K63 N increases to 373.5 (Figure S6(b)). Hydrogen bonds play a crucial role in establishing interactions between protein structural folds and structures, and the number of hydrogen bonds determines the stability of the overall protein structure.^[25]^ Based on changes in the RMSD of Cα and the number of hydrogen bonds, it was speculated that the increased catalytic stability of K63 N may be attributed to the increase in the number of hydrogen bonds within the tetramer stabilizing the overall conformation of the BsADC protein.

**Figure 4:**
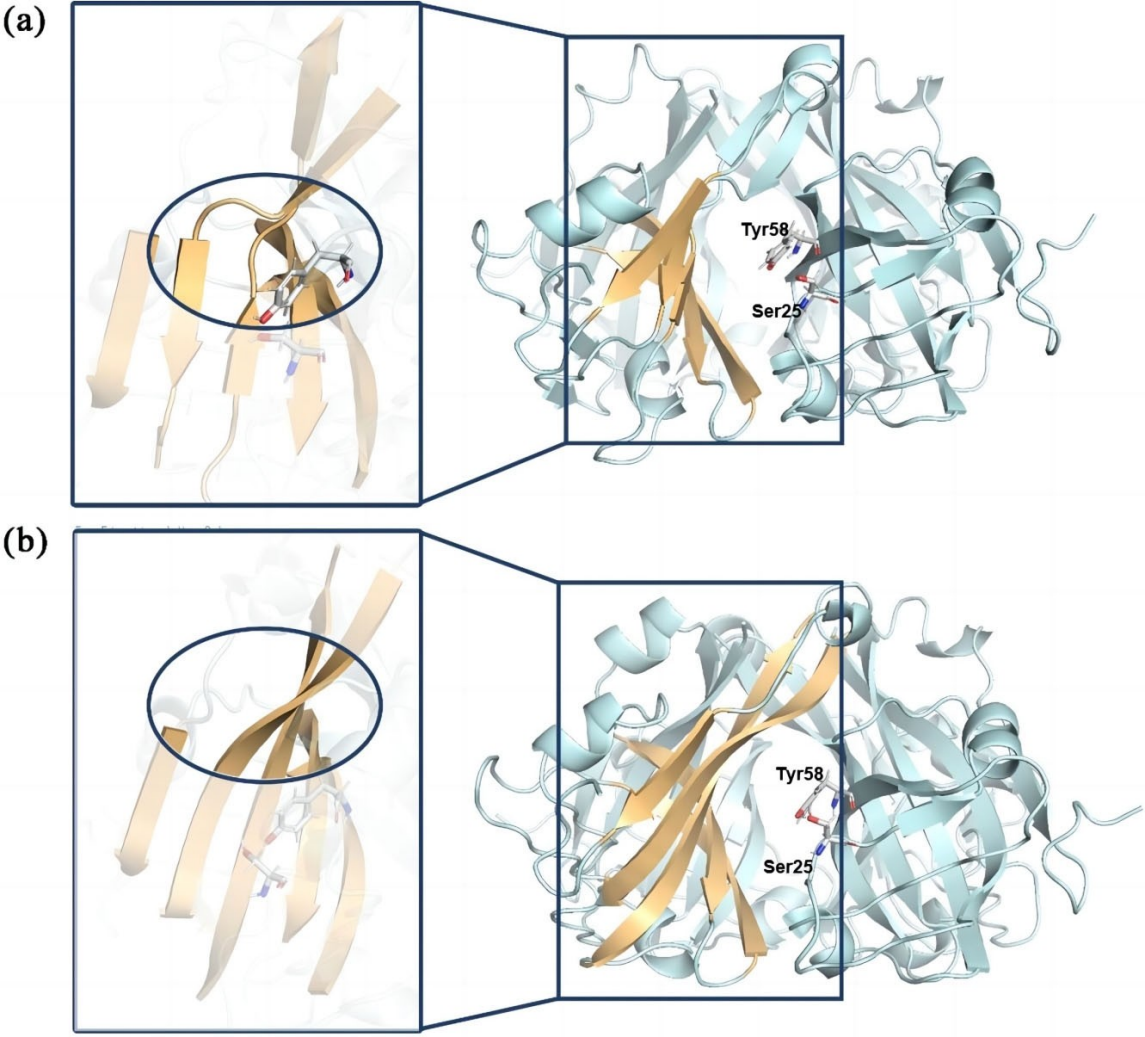
Structure of β‐pleated sheet part of substrate channel. (a) In variant N3, the substrate channel β‐pleated sheet sides β2 and β3 correspond to the regions Y2‐M6, G8‐H11 and I86‐M88, K91‐S94. (b) In the panD‐int, the substrate channel β‐pleated sheet sides β2 and β3 correspond to the regions Y2‐T14 and G82‐S94.

By visualizing molecular dynamics results, it could be found that the adjacent subunit sides in the active pocket region are formed by five parallel β‐pleated sheet structures (Figure 4). These structures are β1 (S104‐L108), β2 (Y2‐T14), β3 (G82‐S94), β4 (N41‐N48), and β5 (G52‐I59), progressing inward from the substrate channel. It was observed that in N3, the substitution of S7 to N situated in β2 induced alterations in the amino acid side‐chain conformation between the N7 side‐chain and I86‐M91 on β3. This resulted in the disruption of the β‐pleated sheet structures of M6‐N7 and S89‐K91, consequently leading to the disturbance of the continuous structure of β2 and β3. To further analyze the impact of these structural changes, RMSF value was calculated (Figure S7), which indicates the degree of flexibility of amino acid residues, with higher RMSF values indicating greater flexibility.^[26]^ It was found that the RMSF value of the folded side of the substrate channel increased, suggesting a decrease in its overall rigidity and thus the flexibility of the entire substrate channel increased. In addition, the molecular docking results revealed an decrease in the binding energy of N3, from −5.042 kcal/mol to −5.17 kcal/mol. Drawing upon the alteration in binding energy^[27]^ and the structural changes observed in the β‐pleated sheet side of the substrate channel, a hypothesis was formulated. It posited that the augmented flexibility of the active pocket promoted the interaction between the active center and the substrate, thereby enhancing catalytic efficiency.

**Figure 5:**
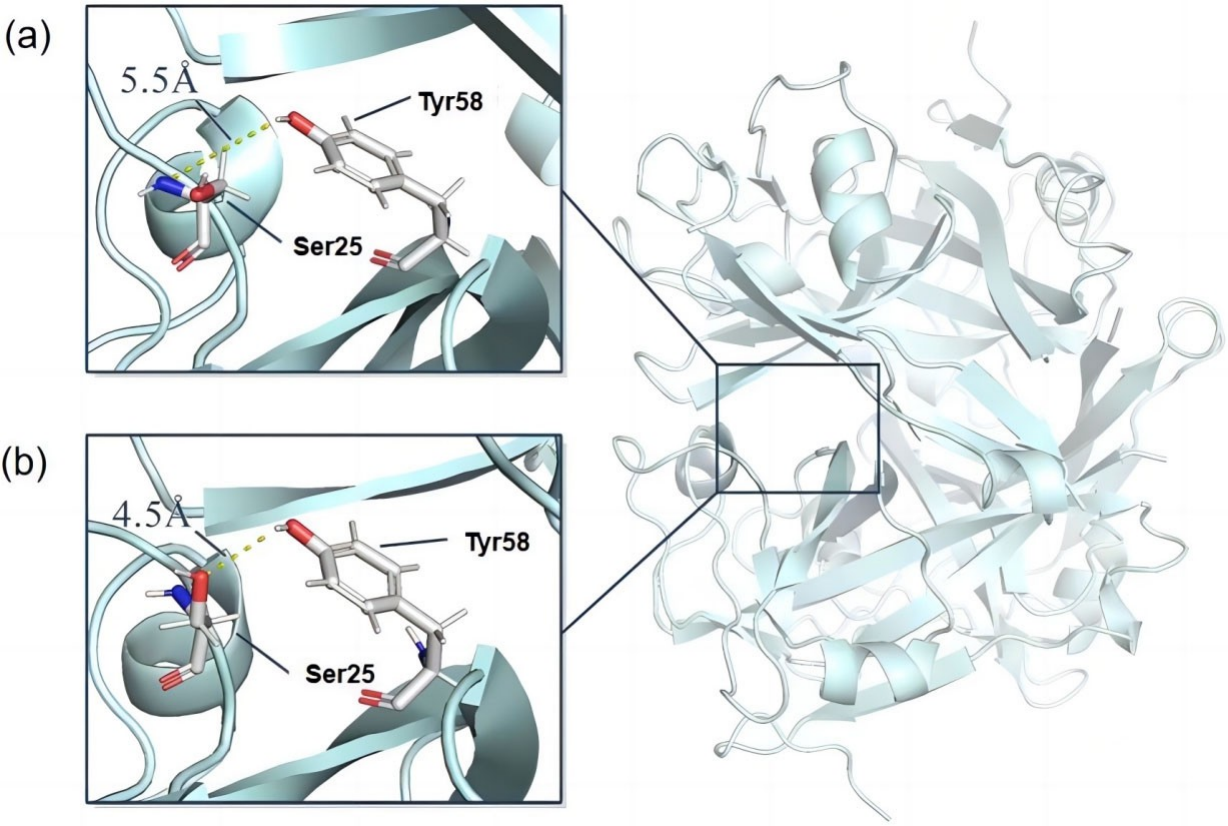
Location of Y58 proton donor and S25 active center in variant N1 and Y1. The yellow dotted line indicates the distance(d1) between S25 and Y58. (a)d1 of Y1. (b)d1 of N3.

Based on the DCCM[^25^, ^26^, ^28^] and RMSF results of single substitutions(Figures S8 and S9), it is speculated that substitutions in S7 and A99 may affect the amino acids near the active pocket. It is worth noting that the distance (d1) between the proton donor Y58 and the active site S25 increased from 3.4 Å to 4.5 Å and 5.5 Å (Figure 5). The increase in d1 has been demonstrated to reduce incorrect events during the catalytic process of BsADC, thereby enhancing catalytic stability. It was inferred that the better catalytic stability of Y1 compared to N3 was mainly attributed to the longer d1, which posed a greater challenge for incorrect protonation. Furthermore, it was found that compared to the wild type, d1 in mutant variants N3 and Y1 were directly influenced by a loop region adjacent to the active pocket surface, consisting of R3‐G8, S94‐P103. This loop region has been reported to frequently alter the structure of the active pocket, thereby improving enzyme performance.^[29]^ Further structure analysis on this loop region in the mutant variants N3 and Y1 discovered that the substitution of residue S7 with a tyrosine or asparagine side chain containing a benzene ring and the A99 substitution to glutamic acid resulted in an increased steric hindrance, diminishing the flexibility of the loop region. Consistent with this, RMSF analysis revealed a reduction in the average RMSF value of the loop region from 0.79 to 0.73, indicating an augmentation in the rigidity of the loop region. Therefore, it was hypothesized that the structure of the loop region could be changed by modifying the steric hindrance of residues, leading to an increased distance between the proton donor and the active site, mitigating mechanism‐based inactivation and thus enhancing catalytic stability.

## 3 Conclusions

In this study, the BsADC variant I88 M/I126* from *Bacillus subtilis* was chosen as the template to construct variant library. By two rounds of screening, four benifical positions that affect the catalytic activity and stability of BsADC were identified. The variant panD‐63 was chosen as the foundation for saturation mutagenesis at the other three sites. By combining substitutions and calculating the folding free energy of the combination, two combinations N3 (S7 N/K63 N/I88 M/A99E/K113R/I126*) and Y1 (S7Y/K63 N/I88 M/A99E/K113R/I126*) were experimentally identified (Figure S10). These two combinations showed improvement in catalytic efficiency, stability and total turnover number. The impacts of substitutions at the four sites on the structure and function of BsADC were elucidated through a combination of hydrophilic analysis of amino acid residues, molecular dynamics analysis, and structural analysis of the two combinations. Enhancing catalytic efficiency can be achieved by elevating the hydrophilicity of crucial amino acid residues within the BsADC active center. This improvement aimed to enhance the binding capacity of BsADC active center‐associated amino acid residues with the substrate aspartic acid. Increasing the hydrophilicity of the outer surface of the protein was proposed as a means to augment enzyme stability in the catalytic environment. By modifying the steric hindrance of the amino acid side chain near the active pocket, the local conformation was adjusted to enhance the flexibility of the active pocket region. This adjustment facilitated the entry of the substrate into the active pocket, promoting binding to the active center and thereby improving catalytic efficiency. Increasing the distance between the proton donor and the active site and stabilizing the conformation around the proton donor aimed to mitigate the mechanistic inactivation phenomenon caused by incorrect protonation, consequently leading to an increase in catalytic stability. This strategy serves as a valuable guide for comprehending the catalytic process mechanism, optimizing enzyme catalytic performance, and designing more efficient ADC variants.

### 3.1 Ethical Approval

This article does not contain any studies with human participants or animals performed by any of the authors.

## 4 Funding

This study was funded by the National Key Research and Development Program of China (grant number 2021YFC2101000). This study was funded by the National Natural Science Foundation of China (grant numbers 22378015 and 52073022).

## 5 Highlights

– Four beneficial positions were identified based on directed evolution campaign.
– The best recombined variants were obtained by computational analysis(1.95‐fold improvement for activity and 1.89‐fold improved for stability).
– Hydrophilicity and steric hindrance within the active center were identified as important influencing factors.

## Author Contributions

Zihan Liu‐Investigation, Methodology, Writing Original Draft; Yiheng Liu‐Methodology; Qixuan Jiang and Haijun Xu‐Conceptualization, Reviewing,‐Luo Liu‐Supervision, Funding acquisition, Reviewing. All authors read and approved the final manuscript.

## Conflict of Interests

The authors have no relevant financial or non‐financial interests to disclose.

6

## Supporting information

As a service to our authors and readers, this journal provides supporting information supplied by the authors. Such materials are peer reviewed and may be re‐organized for online delivery, but are not copy‐edited or typeset. Technical support issues arising from supporting information (other than missing files) should be addressed to the authors.

Supporting Information

## Data Availability

The data presented in this study are available on request from the corresponding author.
