# Supplementary material for: Molecular Engineering L‐Aspartate‐Alpha‐Decarboxylase to Enhance Catalytic Stability and Performance
Source: ChemistryOpen. 2024 Oct 25;14(2):e202400236. doi: 10.1002/open.202400236 (PMC11808261; doi:10.1002/open.202400236)
Supplement: Supplementary file 1 — Supporting Information [file OPEN-14-e202400236-s001.pdf]

ChemistryOpen

Supporting Information

# Molecular Engineering L-Aspartate-Alpha-Decarboxylase to Enhance Catalytic Stability and Performance

Zihan Liu, Yiheng Liu, Qixuan Jiang, Haijun Xu, and Luo Liu*

Table S1 . The sequence of primers for PCR

| Name | 5'-3' sequence |
|---|---|
| T7 | TAATACGACTCACTATAGGG |
| T7-term | TGCTAGTTATTGCTCAGCGG |
| S7 | AATGATGNNKGGAAAACTACACCGCGC |
| A99 | AGAGGCCNNKAGCCACGAACCGAG |
| K113 | ATCAGAATNNKATCGAGCAGATGCTGGGTAAT |

Table S2 Reaction system for random mutation

| Reagents | final concentration | volume | volume |
|---|---|---|---|
| Distilled water | - | (13.2-x)μL | (33-y)μL |
| RandomMut buffer (10X) | 1X | 2μL | 5μL |
| Mutation enhancer (10X) | 1X | 2uL | 5μL |
| dNTP (2.5mM each) | 0.25mM | 2μL | 5μL |
| Template DNA | 0.2pg/μl -20ng/μL | xμL | yμL |
| Primer mix(10μM each) | 0.2μM each | 0.4μL | 1μL |
| RandomMut DNA polymerase | - | 0.4μL | 1μL |

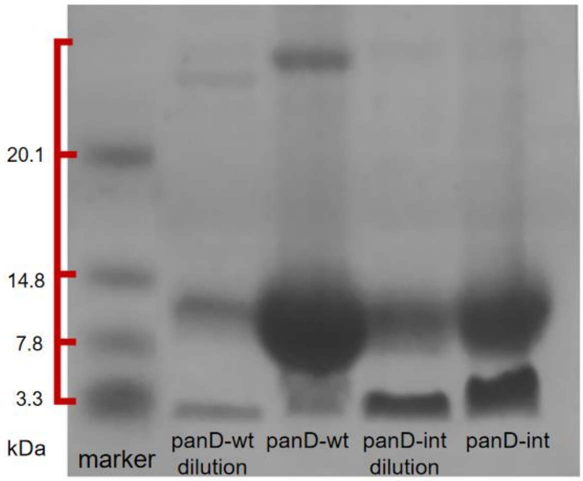


Figure S1 The result of SDS-PAGE. The results showed that the BsADC bands were basically consistent with those reported, and there were no impurity bands.

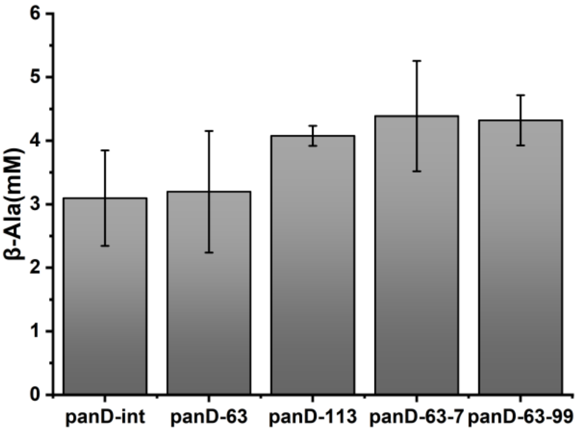


Figure S2 Directed evolution screening results. Screening is based on β-Ala accumulation of variant at 10 min.β-Alanine accumulation of different variants within 60 min.

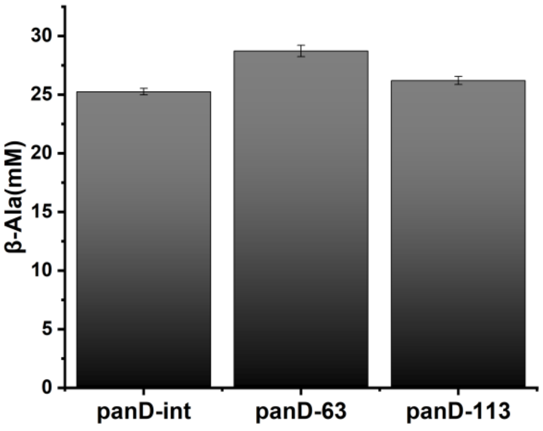

Figure S3 β-Alanine accumulation of different variants within 60 min.

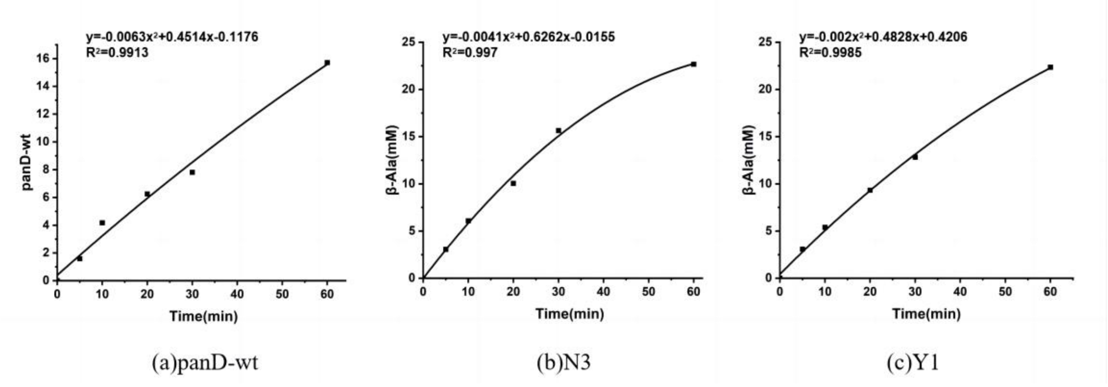


Figure S4 Fitting results of panD-wt, N3 and Y1 β-alanine accumulation

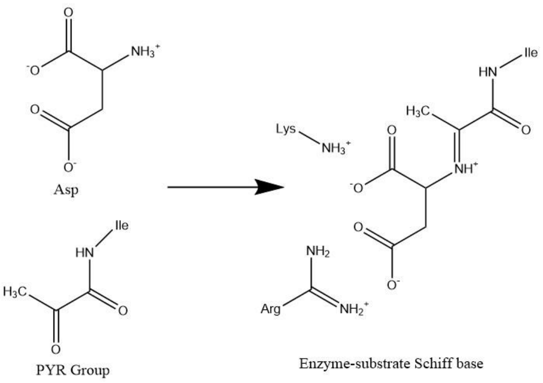


Figure S5 Lys9 bound Schiff base by hydrogen bond.

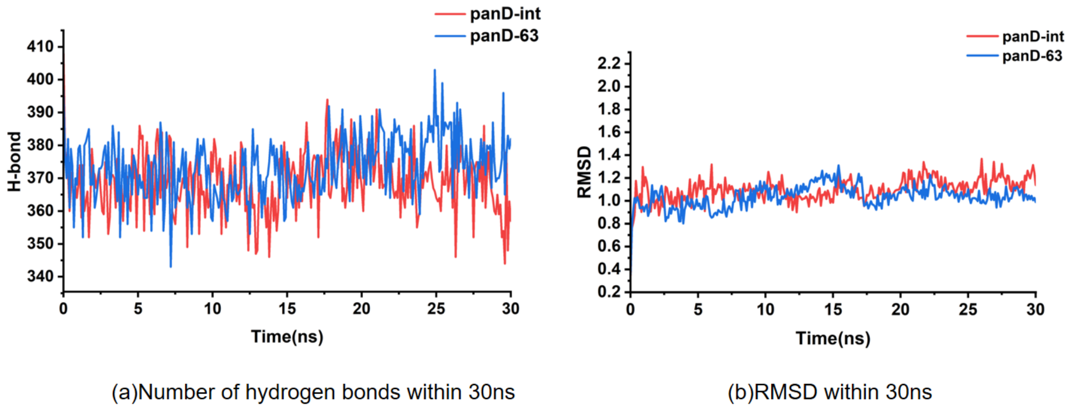


Figure S6 Number of hydrogen bonds and root mean square deviation of BSint and K63N within 30 ns. The average RMSD of K63N decreased to 1.03703, and the number of hydrogen bonds of K63N increases to 373.5.

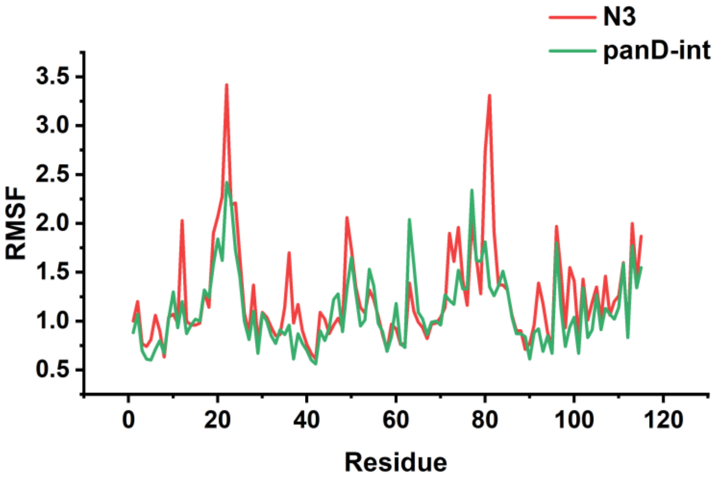


Figure S7 RMSF of panD-int and N3. The RMSF of substrate channel increased.

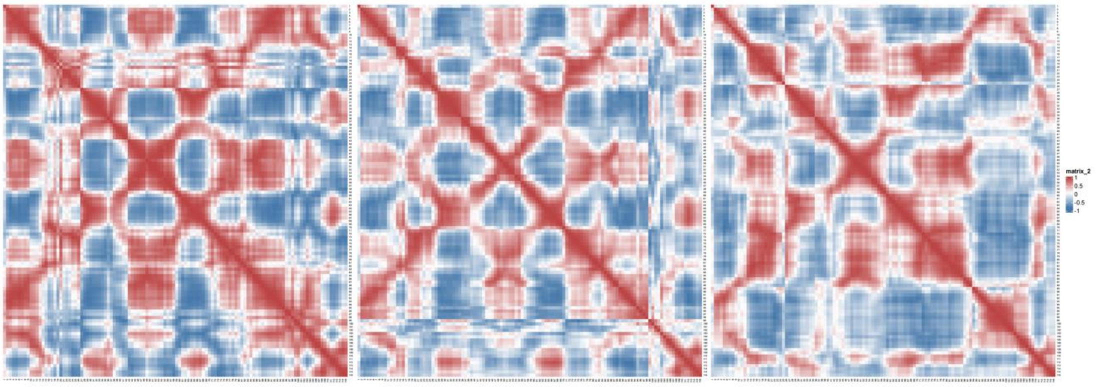

(a)panD-int (b)panD-S7N (c)panD-A99T

Figure S8 Changes of DCCM after mutation. Hiplot was used to obtain insights from the MD trajectories of protein-ligand complexes

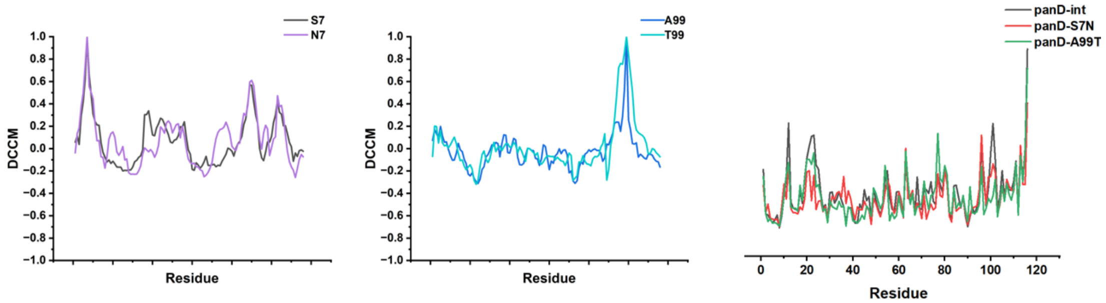


(a)DCCM of site 7 (b)DCCM of site 99 (c)RMSF of BSint、S7N、A99T

Figure S9 (a) Relevant changes in amino acid residues caused by mutation at position 7(b)Relevant changes in amino acid residues caused by mutation at position 99(c)RMSF of panD-int、panD-S7N、panD-A99T.

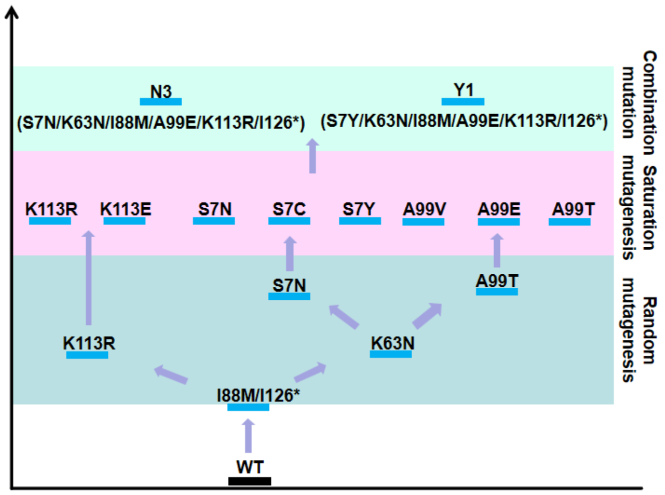


Figure S10 The process of molecular engineering.

Supplementary Note -MD simulation

MD(Molecular dynamics) simulations were conducted using YASARA (Yet Another Scientific Artificial Reality Application) software. The simulation setup included constrained molecular dynamics, with energy minimization and tension release correction performed prior to the simulation. The electrostatic interactions were handled using the PME (Particle Mesh Ewald) method. The simulations were conducted at 101.25 kPa atmospheric pressure, pH 7.4, and a temperature of 298 K, with the protonation states of protein residues modeled under these conditions. The AMBER14 force field and TIP3P (Transferable Intermolecular Potential with 3 Points) water model was employed to construct the system, with the simulation box extending 5 Å from the outermost atoms. The system was neutralized using $Na^+$ and $Cl^–$ ions to achieve a net charge of zero. Trajectory analysis was performed using YASARA's "md_analyze.mcr" macro, with the simulation running on a supercomputing platform or microcomputer. The model's motion was predicted over 30 nanoseconds, and its RMSD (Root-Mean-Square Deviation) was confirmed to have stabilized, with a timestep of 2×1.25 fs.
